# Effects of Manganese Carbonate Addition on the Carbocatalytic Properties of Lignocellulosic Waste for Use in the Degradation of Acetaminophen

**DOI:** 10.3390/polym16233316

**Published:** 2024-11-27

**Authors:** Camila Mosquera-Olano, Carolina Quimbaya, Vanessa Rodríguez, Angie Vanessa-Lasso, Santiago Correa, E. D. C. Castrillón, John Rojas, Yenny P. Ávila-Torres

**Affiliations:** 1 Environmental Remediation and Biocatalysis, Institute of Chemistry, University of Antioquia UdeA, Medellín CP 050010, Colombia; m.mosquera1@udea.edu.co (C.M.-O.); carolina.quimbaya@udea.edu.co (C.Q.); vanessa.lasso@udea.edu.co (A.V.-L.); elkindariocastellon@gmail.com (E.D.C.C.); jhon.rojas@udea.edu.co (J.R.); 2 Chemistry and Biotechnology, Santiago de Cali University, Cali CP 760032, Colombia; vanerp64@yahoo.es; 3 Grupo de Investigación Cecoltec, Cecoltec Services, Medellín CP 050016, Colombia; scorrea@cecoltecservices.com

**Keywords:** revalorization, wastes, acetaminophen

## Abstract

A carbon-based material was synthesized using potato peels (BPP) and banana pseudo-stems (BPS), both of which were modified with manganese to produce BPP-Mn and BPS-Mn, respectively. These materials were assessed for their ability to activate peroxymonosulfate (PMS) in the presence of MnCO_3_ to degrade acetaminophen (ACE), an emerging water contaminant. The materials underwent characterization using spectroscopic, textural, and electrochemical techniques. Manganese served a dual function: enhancing adsorption properties and facilitating the breaking of peroxide bonds. Additionally, carbonate ions played a structural role in the materials, transforming into CO_2_ at high temperatures and thereby increasing material porosity, which improved adsorption capabilities. This presents a notable advantage for materials that have not undergone de-lignification. Among the materials tested, BPS exhibited the highest efficiency in the carbocatalytic degradation of ACE, achieving a synergy index of 1.31 within just 5 min, with 42% ACE degradation in BPS compared to BPS-Mn, which achieved 100% ACE removal through adsorption. Reactive oxygen species such as sulfate, hydroxyl, and superoxide anion radicals were identified as the primary contributors to pollutant degradation. In contrast, no degradation was observed for BPP and BPP-Mn, which is likely linked to the lower lignin content in their precursor material. This work addressed the challenge of revalorizing lignocellulosic waste by highlighting its potential as an oxidant for emerging pollutants. Furthermore, the study demonstrated the coexistence of various reactive oxygen species, confirming the capacity of carbon-based matrices to activate PMS.

## 1. Introduction

Each year, approximately 50 million tons of lignin are generated globally, with 98% to 99% incinerated for the purpose of producing energy and steam in pulp manufacturing facilities. Only a fraction, primarily, is commercially recovered under a biorefinery concept. There are significant opportunities to create high-value products from technical lignin, especially given the versatility of these modified materials in the catalysis industry [1,2]. Generally, when organic matter composed of hemicellulose, cellulose, and lignin is subjected to thermochemical processes such as pyrolysis, carbonaceous materials are generated, characterized by aromatic structures, and oxygenated groups, including aldehydes, ketones, esters, and carboxylic acids. These characteristics endow these materials with the capacity to adsorb molecules classified as emerging contaminants and heavy metals from water through electrostatic interactions, van der Waals forces, and π-stacking, making them an excellent alternative in addressing current environmental challenges [3,4,5]. This characteristic allows for the reuse of the carbonaceous material and contributes to addressing an environmental issue, such as the persistence of organic molecules and heavy metals in wastewater and drinking water. However, contaminants merely move from one matrix to another, and measurements must be implemented to prevent their accumulation. An important strategy is utilizing these pyrolyzed materials as carbon catalysts [6,7].

To address emerging environmental issues of the 21st century, unmarketable potato peels can be further processed and transformed into value-added materials [8]. Potatoes are among the most significant agricultural crops for human consumption, following wheat, rice, and maize, with a production volume of 376 million tons in 2013 [9]. However, the potato industry generates substantial waste—leftover tubers, peels, and raw materials—that accumulate and pose environmental challenges. It is estimated that between 12% and 20% of the total volume of potatoes processed results in peelings and other by-products [10]. Similarly, bananas are widely cultivated and nutritionally valuable crops, grown across more than 130 tropical and subtropical countries. In 2016, India, China, the Philippines, Brazil, and Ecuador were the leading banana producers, with a global yield of approximately 144 million metric tons, highlighting its economic relevance [11]. Despite this, a large portion of the banana plant is discarded as waste, disrupting ecosystems and contributing to ecological hazards. Banana pseudostems, often seen as low-value waste, can also be repurposed into valuable materials, further supporting efforts to mitigate contemporary environmental challenges [12].

Both species represent high-value agroindustrial waste, however, their application in adsorption and/or biocatalytic processes can be influenced by the generation of new physicochemical properties from the pyrolysis process and its lignitic composition. The composition of these residues is shown in Table 1 [13,14].

The characteristics resulting from the pyrolysis process in both species can result in the generation of molecules in a greater or lesser proportion, directly influencing their applications. For example, the greater amount of lignin in banana pseudostem results in a greater production of phenols and aromatic hydrocarbons, which are byproducts of lignin decomposition [15]. Currently, several studies have developed similar materials with environmental applications. An example is the work of Grisales et al. [16], who produced biochar derived from palm fiber residues, in combination with H_2_O_2_ and O_5_S^−2^, in the removal of drugs such as valsartan, acetaminophen, and cephalexin in water. Similarly, Quimbaya et al. [17] produced a carbonaceous material derived from industrial waste, specifically sawdust, which was modified with manganese to form an improved biochar. This material was used to activate PMS by ultrasound (US) at different frequencies, achieving the degradation of ciprofloxacin in water. Demonstrating that biochars can be optimized by modifications with transition metals and whose improvement potential depends on the molecular composition of the species in their cell walls, in addition to these methods, the transcendental value and carbocatalytic power of the waste are demonstrated even by incorporating an oxidizing agent in the system.

In this context, it is necessary to add oxidizing agents to the carbonaceous material that are activated by electron transfer, allowing the decomposition and even elimination of the contaminant. Thus, various catalytic carbons have been synthesized that activate inorganic peroxides (hydrogen peroxide, peroxymonosulfate, and peroxydisulfate), generating radical species (^•^HO, ^•^SO_4_^−^) and non-radical species (^1^O_2_) after the homolytic bond dissociation of the peroxide (O-O) bond. These species are capable of degrading persistent molecules such as antibiotics (β-lactam antimicrobials, sulfa drugs, 4-aminobenzoic acid, and tetracycline-class compounds) and antipyretics like acetaminophen, among others [18], Equations (1)–(6).

(1) $$-COOH+{HSO_{5}}^{-}\to {SO_{4}}^{•-}+H_{2}O+-COO^{•}$$

(2) $$-OH+{HSO_{5}}^{-}\to {SO_{4}}^{•-}+H_{2}O+-O^{•}$$

(3) $$\;\;\;defect+{HSO_{5}}^{-}\to {SO_{4}}^{•-}+{OH}^{-}+{defect}^{+}$$

(4) $${HSO_{5}}^{-}+H_{2}O\;\to \;{SO_{4}}^{2-}+2^{•}OH\;+\;H^{+}$$

(5) $${HSO_{5}}^{-}\;+\;{SO_{5}}^{2-}\;\to \;{HSO_{4}}^{-}\;+\;{SO_{4}}^{•-}\;{+\;}^{1}O_{2}$$

(6) $${HSO_{5}}^{-}+H_{2}O_{2}\;\to \;H^{+}\;+\;{SO_{4}}^{2-}\;+\;H_{2}O\;{+\;}^{1}O_{2}$$

ACE is classified as an emerging contaminant. These are chemical substances environmentally poorly regulated, with adverse effects on human health, which are not fully known. Personal care products, pharmaceutically active compounds, and endocrine-disrupting chemicals are among the most popular CECs. Many CECs are bio-accumulable and recalcitrant to conventional processes in wastewater or drinking water. The ACE can induce adverse physiological effects at low concentrations in humans and animals. Numerous studies worldwide have reported its presence in various aquatic environments (200 ng/L), raising concerns about the potential negative impacts on water quality, human health, and wildlife. The ACE generates intermediates depending on pH. Its degradation products (hydroquinone and benzoquinone, among others) and intermediates present significant challenges for effective monitoring, detection, and the development of suitable treatment technologies. Membrane separation and third treatments as oxidation advance the process are options to contribute to this problem [19,20]. Various materials have been developed from carbon-based substances for the degradation of ACE, frequently incorporating nitrogen- and phosphorus-doped carbon nitrides activated through photocatalysis [21,22]. Furthermore, oxidizing agents like periodate and peroxydisulfate have also been utilized. Reactive oxygen species have been produced in every instance, resulting in significant acetaminophen degradation rates. However, there is limited research on the role of transition metals in cleaving the peroxide bond within a carbonaceous matrix, particularly when carbonate ions are included in the process. These ions enhance surface area and generate carbonate radicals, which act as additional active species for the oxidation of organic compounds, Table 2.

On the other hand, an advantage of the PMS is its ease of forming coordination bonds with transition metals, which in turn bond with the carbonaceous matrix, facilitating the cleavage of the double bond. On the other hand, due to its high redox potential, it allows it to oxidize metal oxides, which in turn performs a direct oxidation to the contaminant. Equations (7)–(9).

(7) $$\equiv Mn\left(III\right)-\left(O\right)OS{O_{3}}^{-}+H_{2}O\to \equiv Mn\left(IV\right)-OH+{{SO}_{4}}^{-•}+{OH}^{-}$$

(8) $$HS{O_{5}}^{-}+M^{n+}\to M^{\left(n+1\right)+}+{SO_{4}}^{•-}+{OH}^{-}$$

(9) $$HS{O_{5}}^{-}+M^{n+}\to M^{\left(n+1\right)+}+{SO_{4}}^{2-}+HO^{•}$$

Considering that PMS can be activated by carbonaceous matrices and transition metals, it is expected that the combined effect of these two factors improves performance in removing recalcitrant molecules from aqueous solutions. In this study, banana pseudo-stem, and potato peel were used as sources of lignocellulosic materials. These materials were then thermally exfoliated at 500 °C and subsequently modified with manganese to create a carbon catalyst capable of completely degrading acetaminophen. The nature of raw carbonaceous materials was evaluated to comprehend its implications in the synergy phenomena for the breakdown of organic pollutants. Finally, to demonstrate how lignocellulosic waste can be used to activate PMS. The role of transition metals and their carbonate salts in the process, as well as the structural implications for degrading ACE via reactive oxygen species. This is shown using electrochemical techniques to evidence the degradation and the material’s capacity to transfer electrons.

## 2. Materials and Methods

### 2.1. Reagents

Acetaminophen (marca) was obtained from Research Pharmaceuticals. Acetonitrile (C_2_H_3_N), formic acid (CH_2_O_2_), hydrochloric acid (HCl), hydrogen peroxide (H_2_O_2_), manganese carbonate (MnCO_3_), methanol (C_2_H_6_O), sulfuric acid (H_2_SO_4_), sodium azide (NaN_3_), sodium hydroxide (NaOH), sulfuric acid (H_2_SO_4_), sodium sulfide (Na_2_SO_3_), and p-benzoquinone (C_6_H_4_O_2_) were supplied by Merck, Rahway, NJ, USA. Oxone (KHSO_5_ · 0.5 KHSO_4_ · 0.5 K_2_SO_4_), serving as the source of potassium peroxymonosulfate (PMS, KHSO_5_), was obtained from Sigma-Aldrich, Burlington, MA, USA.

### 2.2. Methods

#### 2.2.1. Carbonaceous Compound Development

Pseudo stem (**PS**) was retrieved from a Tumaco Nariño, Colombia. Thirty grams of sample were subjected to pyrolysis under a nitrogen atmosphere with a constant flow of 124.8 mL per minute at 500 °C in a Mueller & Krempel muffle furnace for 2 h, producing the carbonaceous material (**BPS**). The selected temperature allows for the highest degradation of lignin, with a maximum weight loss rate [27]. This BPS was then reacted with MnCO_3_ at a mass ratio of 1:1 for 12 h. Subsequently, the reaction product was heated to 800 °C (O_2_/N_2_ (20/80)), and for 1 h, generating a manganese-modified carbonaceous material (**BPS-Mn**). Finally, the BPS-Mn was washed with 0.01 M HCl until reaching a pH of 6.5–7.5 and dried at 105 °C for 24 h.

The potato peel (**PP**) waste was sourced from the municipality of Pasto Nariño, Colombia’s main potato-producing region. The treatment followed the same conditions previously described for the pseudostem to obtain **BPP** and **BPP-Mn**.

To determine the lignin content in the raw materials of banana pseudostem and potato peel, a delignification process was carried out using an alkali solution (0.01 M NaOH) for 24 h, followed by drying and characterization with SEM/EDS spectroscopy.

#### 2.2.2. Reaction Mechanisms

Carbocatalysis was performed in a batch flocculation reaction system with a 500 mL sample capacity. The reaction setup was based on previously published results by our group, which define the optimal proportions of catalyst and PMS. Accordingly, 30.6 μM of ACE, 0.5 mM peroxide, and 0.2 g/L of the catalyst (BPS-Mn and BPP-Mn) were added. Finally, adsorption measurements and temperature-induced exfoliation were conducted as control assessments for the carbocatalysts.

Apart from testing the initial and final pH, irrigation water was spiked as a real matrix with the contaminants described above to evaluate the system’s reusability over three consecutive cycles and its overall viability. To identify ROS production, scavengers like tert-butanol and methanol were employed to detect species such as HO^•^ and SO_4_^•−^, respectively. Additionally, sodium azide and *p*-benzoquinone were used to identify O_2_^•−^ and ^1^O_2_. The percentage removal (%R) was calculated for each system, allowing for the final calculation of the synergy index (SI) of the materials in the process. Equation (10):

SI = %Rcarbocatalysis/(%RPMS + %RM)(10)

%RPMS = Degradation using PMS, %RM = Remotion with adsorption, %Rcarbocatalysis = Remotion using carbocatalysis (PMS + material).

### 2.3. Analyses

#### 2.3.1. Chromatography-Based Analysis

The analysis of pollutant evolution was conducted on a UHPLC Thermo-Scientific (Waltham, MA, USA) Dionex UltiMate 3000, fitted with an Acclaim™ 120 RP C18 column (5 μm, 4.6 × 150 mm). For ACE chromatographic analysis, a solvent mixture of acetonitrile and formic acid in a 30:70 (% *v*/*v*) ratio was maintained at a flow rate of 0.5 mL/min, with a detection wavelength set at 230 nm.

#### 2.3.2. Spectroscopic and Electrochemical Characterization for BPS, BPP, BPS-Mn, BPP-Mn Materials

To determine the surface area and pore characteristics, BET analysis was conducted using a Quatachrome NOVA 1200e surface area and pore size analyzer (Boynton Beach, FL, USA). The textural properties of the carbonaceous material will be determined by nitrogen adsorption at −196 °C using an ASAP 2020 Micrometry instrument. The specific surface area (SBET) shall be determined by applying the Brunauer-Emmett-Teller (BET) equation to the isotherm. In addition, the total pore volume (VTP) will be determined, which will correspond to the volume of liquid nitrogen adsorbed at relative pressure (P/Po) of 0.98. Micropore volume (VμP) and external surface area (SEXT) shall be determined using the t-Plot method. The external volume (VEXT) will be calculated using the difference between VTP and VμP. The average pore diameter (DAP) will be calculated using the 4VTP/SBET ratio. The surface morphology of the materials will be examined using scanning electron microscopy (SEM), using a JEOL JSM-6490LV model, along with an energy dispersive spectrometer COXEM (Daejeon, Republic of Korea) (EDS detector EDAX) from Oxford Instruments (Abingdon, UK) to identify the relative content of the elements. Structural defects were evaluated with a Horiba Yvon Jobin dispersive micro-Raman spectrophotometer, Horiba, Ltd., Kyoto, Japan. Dynamic light scattering (DLS) analyses were performed with a Malvern Zetasizer Pro analyzer (Malvern, UK) to assess particle size. Crystal structure patterns were acquired on an XRD-AERIS Diffractometer packed with a High score software Plus version 4.8 (Malvern Panalytical, Almelo, The Netherlands), (COXEM, Daejeon, Republic of Korea) with EDAX from Oxford Instruments, England. X-ray diffraction was used to determine the characteristic diffraction planes of crystalline phases, including both graphite and those corresponding to manganese carbonate and metal oxides. Electrochemical Impedance Spectroscopy (EIS). Cyclic voltammetry (CV) was carried out using a PalmSens4 electrochemical station coupled with a conventional three-electrode system consisting of a working electrode with the material, an Ir counter electrode, and an Ag/AgCl/KCl reference electrode. Each working electrode was prepared by an impregnation method on FTO-coated glass slides (30 mm × 20 mm × 2 mm), supplied by TechInstro (New Delhi, India). Before each experiment, FTO sheets were soaked in an HOCl_4_ solution (1 × 10^−3^ M) for 1 h. Each material (0.2 g) was suspended in 10 mL of water, and the FTO sheet was immersed in each suspension for 30 s and then dried using a spin-coater. This process was repeated five times to achieve a complete FTO coating. Finally, the FTO-coated sheets were heated in a gas-controlled oven at 500 °C for 60 min repeating the procedure twice to ensure thorough material deposition on the FTO sheets.

## 3. Results

### 3.1. Spectroscopic and Textural Characterization

**Figure 1:**
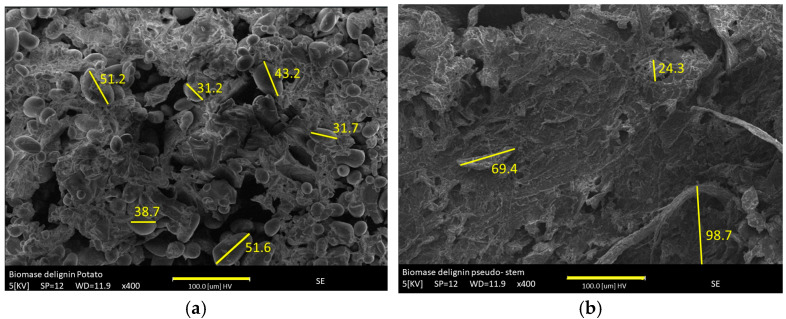
(**a**). PP and (**b**). PP without lignin using alkali treatment.

Table 3 lists the textural properties of raw materials and their modified materials. Raw materials exhibited the less porous structure as the lowest total volume, but PS showed a major micropore structure and a larger specific surface area (23.3 m^2^/g). The carbonization process at 500 °C acted mainly on the cellulose and hemicellulose units depolymerizing them into smaller units resulting in a large carbon-rich mass, mesopore structure, and wider micropore width. Conversely, PS with a larger lignin content upon pyrolysis rendered a more micropore structure although not reflected on the specific surface area. This result was confirmed by performing the material delignification process with NaOH. The lignin content was PS = 59.48% and PP = 20.06%. It was also evidenced in the SEM micrographs of the raw materials without lignin, showing how the raw potato presents an orderly arranged cell wall with a structure surrounded by starch granules. In contrast, the pseudo exhibited rod structure with a relatively smooth, clean, compact surface without lignin, Figure 1a,b.

Interestingly, the manganese-modified materials showed the largest pore volume, micropore amount, and specific surface area irrespective of the raw material source. This phenomenon is explained by the interaction of manganese metal with the planar aromatic structures of the biochar which become stronger upon treatment at 800C. This temperature might generate CO_2_ microbubbles which destroy the heterogeneous surface of the carbonoceus material forming a prevalent reticulate micropore structure of smaller pore width.

**Figure 2:**
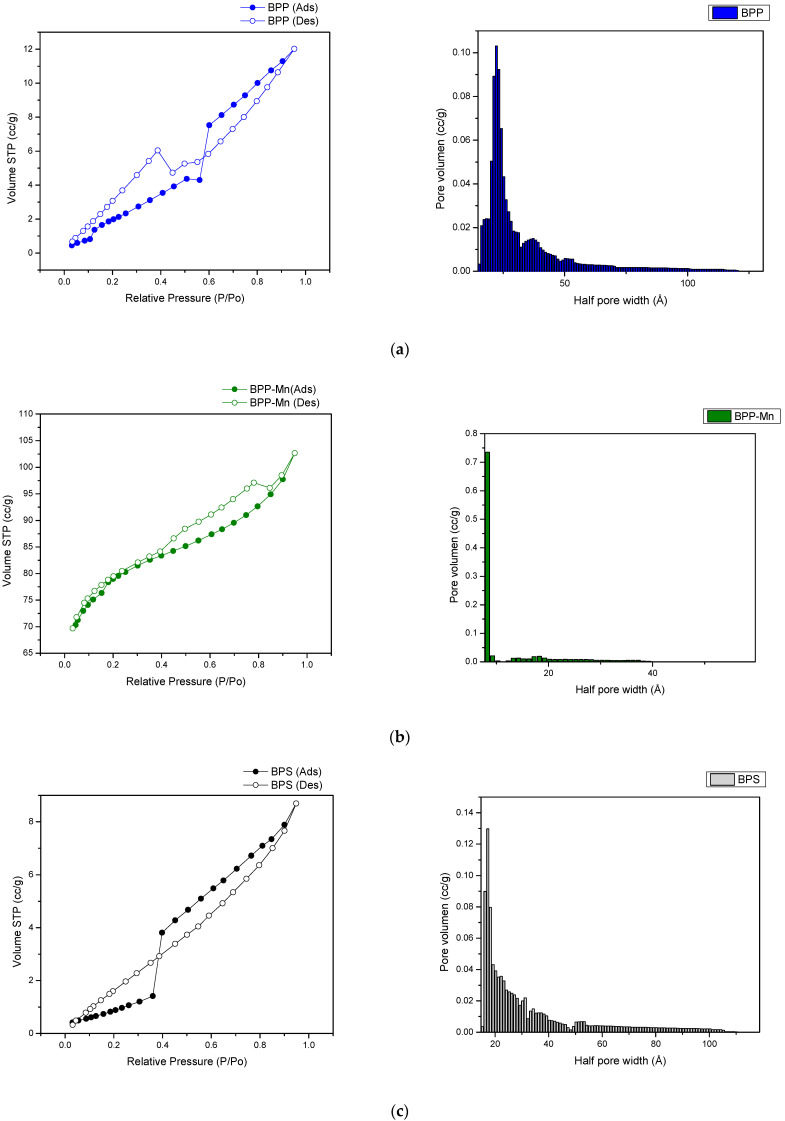

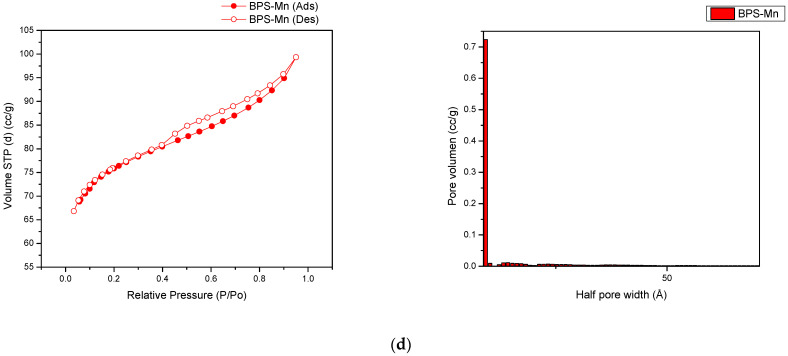
BET adsorption-desorption and size pore distribution for (**a**). BPP, (**b**). BPP-Mn, (**c**). BPS, (**d**). BPS-Mn.

To further study the effect of manganese cation on the microstructure of the **BPP** and **BPS**, nitrogen-adsorption-desorption experiments were conducted and the results are displayed in Figure 2a–d along with the respective pore size distribution plots. The hysteresis loop analysis rendered a wedge-shaped pore or **H3** isotherm for the manganese-modified materials. This pore shape is formed by the loose stacking of flaky particles. Irrespective of whether the parent carbon material is graphitized or non-graphitized, the confined spaces between the graphene stacks tend to be wedge-shaped. The steep increase in the amount adsorbed at very low pressure (P/P_0_ < 0.05) is mainly due to the filling of the micropores. After this initial sharp rise, adsorption continues in mesopores and macropores and the amount adsorbed increases gradually as pressure approaches saturation vapor pressure. There is a parallel hysteresis branch over the reduced pressure range between 0.4 and 1, which is different from the vertical hysteresis branch observed in mesoporous solids with cylindrical pores. This phenomenon is typical of the majority of adsorption isotherms reported in the literature for other adsorbates on activated carbon and activated carbon fibre [28].

Further, manganese-modified materials showed a more left-skwewed distribution indicating a more prevalent micropore structure from 8 to 9 nm in pore width having a predominant wedge shape as described above. On the other hand, at a fisrt glance, the sorption isotherms of carbonized materials resemble a type II isotherm where at low relative pressures have a steady increase reaching an infleccion point where the monolayer is completed and a multilayer sorption get started. However, the desorption region depicts a disticnt behavior where at high relative pressures some pore shrinking occurs followed by pore cavitation in the micropore region. This behavior is more common in generalized sigmoidal isotherms. Thus, hysteresis is caused by the geometries of the vapor–liquid interface of the partially filled pores. Two different geometries are possible for the same vapor pressure only depending on the history of their formation. The change of geometry, which can be generally interpreted as a relaxation process, is irreversible but after all a thermodynamical equilibrium is achieved both in the adsorption and desorption branch [29].

**Figure 3:**
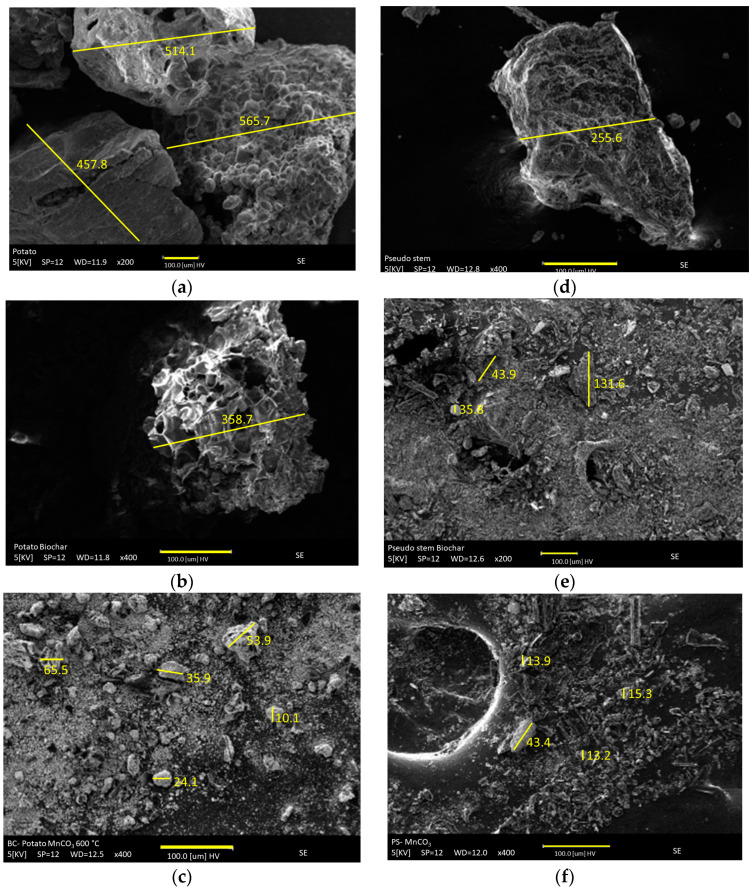
SEM- images of raw materials, carbonaceous materials, exfoliated and modified with manganese. (**a**). PP, (**b**). BPP, (**c**). BPP-Mn, (**d**) PS, (**e**). BPS, (**f**). BPS-Mn.

Figure 3a–d Shows the microstructure and morphology of the raw materials and modified materials. The particle size becomes smaller as it is thermally exfoliated and modified with manganese. The metal salt used in the synthesis plays an important role because the carbonate moiety can oxidize to CO_2_, and in turn, these gas molecules affect the grain size and, consequently, the porosity of the material. Additionally, the water adsorbed on the material surface could be influenced by the acid-base equilibrium of carbonate vs. bicarbonate, enhancing the effect of thermal exfoliation. From that point on, the materials with the largest surface area (BPP, BPP-Mn, BPS, and BPS-Mn) were chosen to establish the role of the starting material on the stabilization and properties of the pyrolyzed material, as well as its modification with manganese. Manganese materials seem to have more coating on the surface of the raw materials since it is not visible in the deep valleys of the surface.

**Figure 4:**
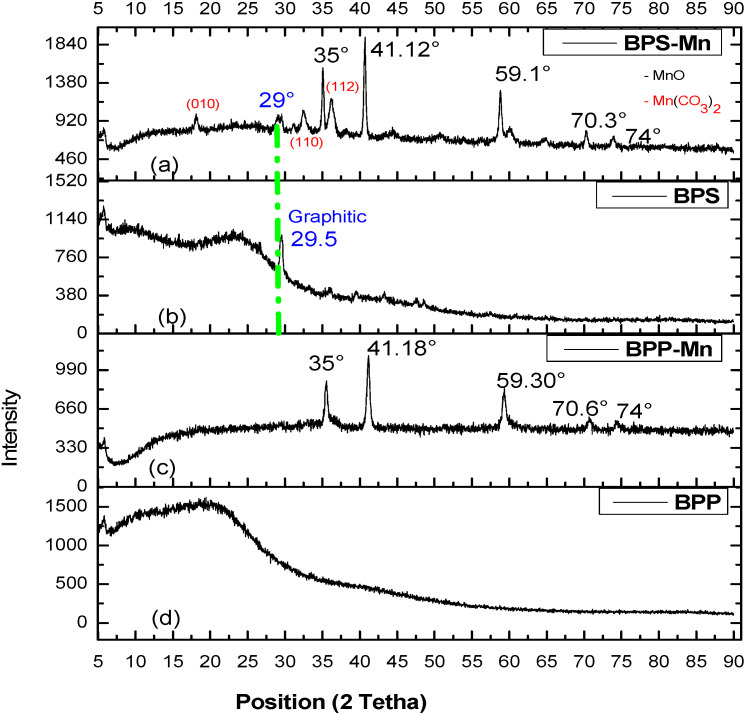
XRD patterns for (**a**). BPS-Mn, (**b**). BPS, (**c**). BPP-Mn, (**d**). BPP. The green dotted line represents Graphitic phase.

In Figure 4, additionally, the XRD patterns for the carbonaceous materials were characterized. A peak is detected at approximately 29° in 2θ, associated with the graphite structure (graphite 002). This peak indicates an arrangement of aromatic layers, and the broadening is due to the small size of the crystallites that are perpendicular to these layers. Meanwhile, the XRD patterns peak at ~35°, ~41°, ~59°, ~70°, and ~74° about the (111), (200), (220), (311), and (222) crystal planes of the MnO phase [29], as well as carbonate manganese (011) 21°, (110) 33° and (112) 36° [30] which observed for BPS-Mn. However, the BPP-Mn material presented high a ratio for MnO phase.

In the Table 4 presents the Charge Capability (CC) measured for the dynamic scattering of light on biochars and manganese-modified materials. The contact with the surface is opposite for precursors, which can be associated with nature. The result is negative for BPS and positive for BPP. These conditions might influence the material properties as adsorbents or carbocatalysts. The manganese-modified materials present a high manganese content, which could be in the form of carbonate and oxide manganese for BPS-Mn or manganese species present in the raw material.

### 3.2. Adsorption and Carbocatalytic Processes

**Figure 5:**
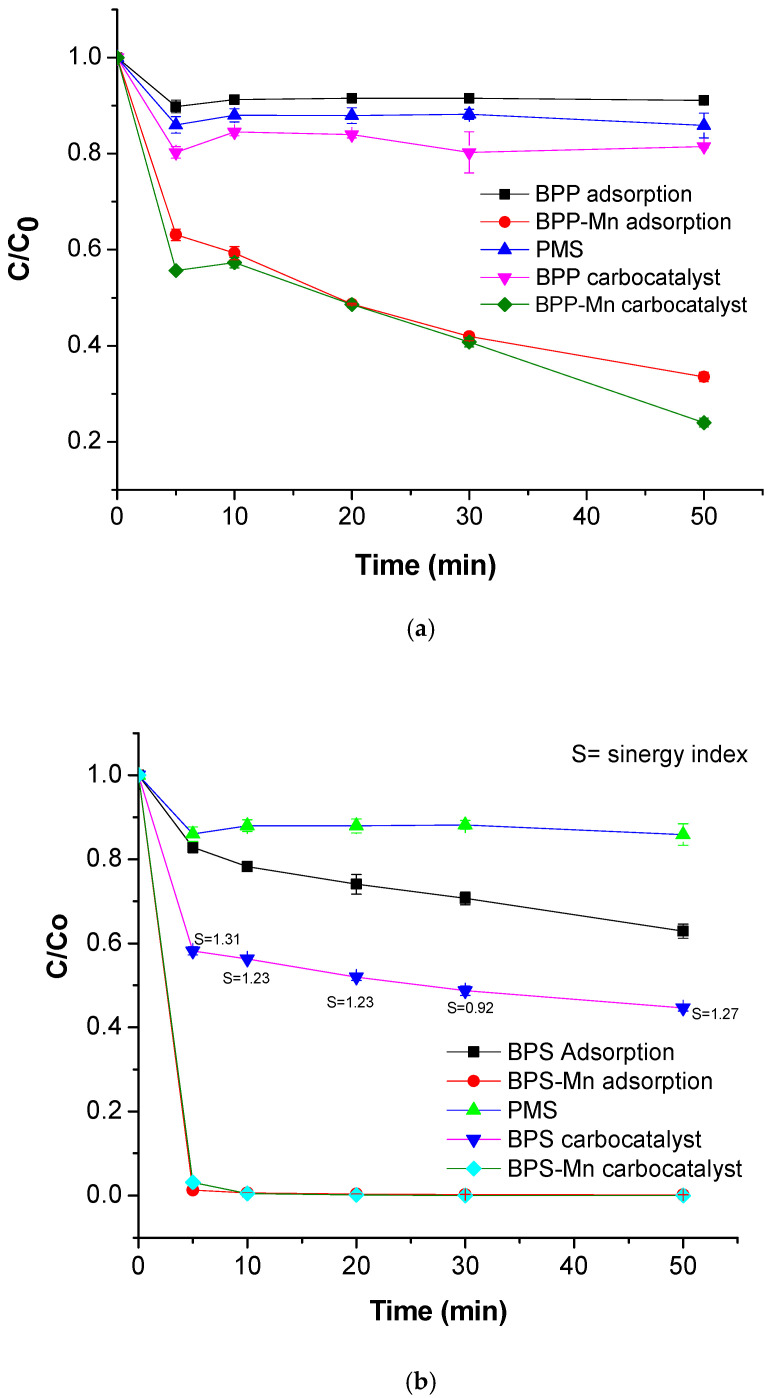
Remotion of ACE with carbonaceous material, (**a**). BPP and BPP-Mn, (**b**). BPS and BPS-Mn.

Figure 5 depicts the adsorption and carbocatalytic process for pyrolyzed and manganese-modified materials. The molecule used for this study was acetaminophen, an emergent contaminant found in hospital wastewaters. The PMS representing an oxidant molecule showed a degradation percentage of 17%. However, the BPP and BPP-Mn behavior suggest these materials had a high adsorption properties. The role of manganese in BPP increases the interaction with the ACE (BPP-Mn remotion of 72%, t = 50 min), Figure 4a. On the other hand, in Figure 4b the pyrolyzed material of pseudo-steam enhances the adsorption vs. peel potato in a special case for BPS-Mn. For the BPS-Mn, the adsorption capacity was ~100% within 10 min of treatment. Further, the chromatographic profile for BPS-Mn was analyzed, finding the presence of degradation products at short treatment times and a high adsorption capacity, Figure S1. This is attributed to competition between processes. Finally, an interesting synergistic effect was appreciated, the BPS material might be activating PMS and degrading the contaminant, increasing the remotion to ~40%. The S-index describes the mathematical additive effect of two independent processes (adsorption and degradation using PMS), being ~1.2, at t = 5 min. Both materials (BPS and BPP) were synthesized under the same experimental conditions. It suggests that the substrate or the liglignocellulosic material defined their adsorbent or carbocatalytic properties. As observed in Figure 1 and the reported lignin content, the presence of lignin in the banana pseudostem enhances the surface area obtained after the pyrolysis process. In contrast, the starch structure in the potato hinders drug adsorption for ACE, a molecule charged negatively to pH circumneutral.

Table 5 illustrates the variations in pH before and after treatment with all materials. The pH parameter plays a significant role in assessing the responsiveness of PMS. In the case of BPS, the basicity is maintained after treatment, indicating a potential negative surface charge during the reaction in the solution. Acetaminophen can undergo deprotonation after treatment, enabling it to coexist in solution with its degradation product. Also, under these conditions PMS can be deprotonated, which may enhance its activation, as it will be analyzed in the proposed subsequent mechanism.

### 3.3. Electrochemical Study on Carbocatalysis Performance

**Figure 6:**
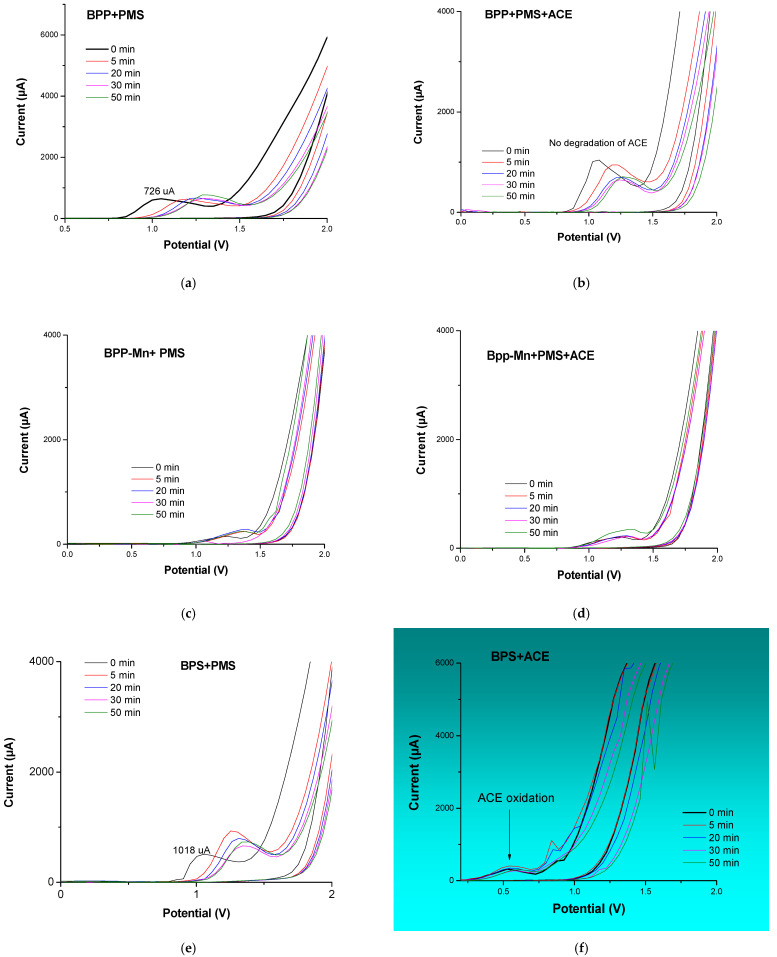

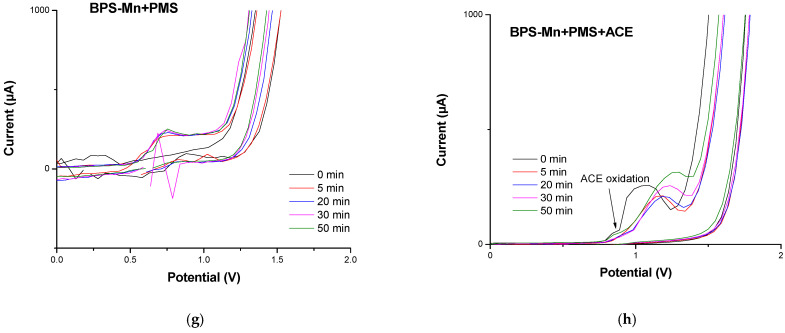
Voltammetry cycles for material activation and carbocatalytic process with and without PMS, scanning rate of 20 mV s^−1^, −2.0 V, and +2.0 V as support electrolyte: [Na_2_SO_4_] = 0.1 M, (**a**). BPP + PMS, (**b**). BPP + PMS + ACE, (**c**). BPP^_^ Mn+ PMS, (**d**). BPP^_^ Mn + PMS + ACE, (**e**). BPS + PMS, (**f**). BPS + PMS + ACE, (**g**). BPS^_^ Mn + PMS + ACE and (**h**). BPS ^_^Mn + PMS + ACE.

**Figure 7:**
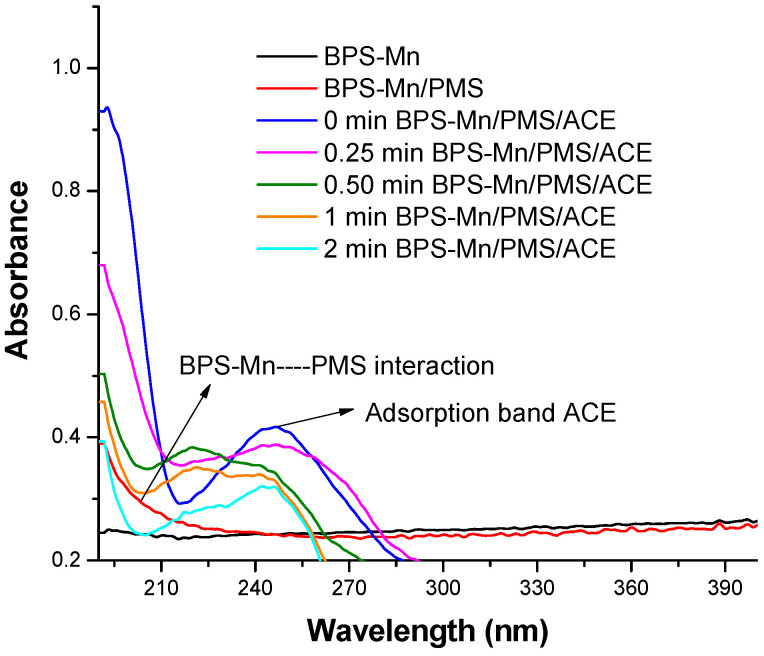
Electronic spectra corresponding to the interaction between ACE-PMS-BPS-Mn for t = 0 min–2 min.

In Figure 6a–h the voltammograms for the materials were recorded under the influence of the oxidizing agent PMS and their exposure as a system to acetaminophen. Oxidation peaks are found at around ∼1.2 V in the presence of PMS and the absence of ACE. The anodic peak (Ia) observed for BPP, Figure 6a indicates differences like the easily oxidizable compounds in the presence of PMS at t = 0 min. The Ia peak can be attributed to vanillin (a lignin degradation product detected around 500 °C) identified in BPP [31] and BPS, Figure 6e. In this case, the intensity peak is major (1018 μA), suggesting more concentration of lignin as a precursor in BPS. For systems with manganese without ACE, the peak (Ia) shifts to higher potential values (1.4 V), highlighting the presence of degradation products formed between 500 °C and 800 °C, Figure 6c,g. The increase in temperature during synthesis and the role of manganese alters the surface properties and consequently impacts the electrochemical oxidation. In the case of the voltammograms corresponding to the systems with the contaminant (ACE) Figure 6f, only an additional oxidation peak is observed for the BPS material, which is consistent with the oxidation of acetaminophen and carbocatalysis for this system about the others, Figure 6b,d,h. The new oxidation peaks are observed for BPS-Mn, which is expected for carbonate and manganese oxide oxidation products. However, these species would be coordinated to ACE, blocking its oxidation for ACE or competition for adsorption places in BPP. It is interesting to note that ACE degradation is also observed for BPS-Mn, as evidenced electrochemically. This is consistent with the reported degradation and its competition with adsorption processes. It is demonstrated that the material, in the presence of manganese, can activate PMS, which in turn interacts with the ACE molecule, facilitating its degradation. To understand this process, absorption spectra were obtained at the characteristic wavelength of acetaminophen, showing an increase in intensity upon interaction with PMS, confirming their interaction, Figure 7. Similarly, experiments were conducted with the carbonaceous material BPS-Mn, with the addition of PMS and ACE, resulting in an increased absorption intensity, which suggests an interaction among these three molecules. The absorption was following since t = 0 min until t = 2 min, observing degradation ACE, as is observed in the electrochemical studies previosly.

### 3.4. Mechanism Elucidation of ACE Degradation with Carbocatalyst BPS

**Figure 8:**
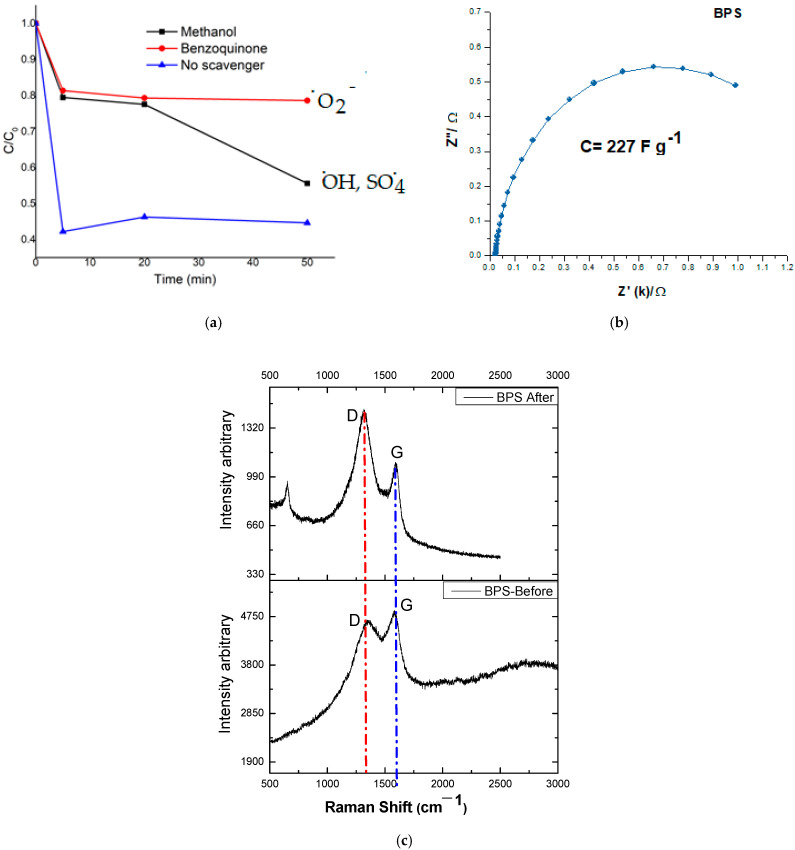
Mechanism elucidation for BPS material (**a**) using scavengers for hydroxyl radical, sulfate radical, and singlet oxygen, (**b**) Electrochemical impedance for BPS, (**c**) and RAMAN shift for BPS after/before treatment. The blue dotted line represents Graphitic phase. The red dotted line represents Graphitic phaseGraphitic structure Diamond structure.

The molecules that scavenge hydroxyl, sulfate, and anion radical superoxide were tested in the degradation of acetaminophen. They were blocking the degradation kinetics by competition with the scavenger allowed for identifying the species responsible for removing acetaminophen, as shown in Figure 8a. On the other hand, the electrochemical impedance study of the BPS material was conducted to demonstrate its capacitive capabilities, which allow for better electronic transfer in this type of carbonaceous material, with a value of 227 F/g, placing it within the range for materials with a high degree of lignin, as shown in Figure 8b. Finally, RAMAN analyses were performed before and after using the material, finding a relationship between the exposed surface functional groups and their role in the degradation process, Figure 8c.

## 4. Discussion

In Figure 2a–d the morphological change of the raw material compared to the modified material is observed. The mesoporosity increases due to temperature. The gases generated during the pyrolysis process promote thermal exfoliation. However, it is notable that the metal decreases mesoporosity due to an increase in its width and volume (Table 3). This may be related to the ease with which the carbonate in the metal salt generates CO_2_ and its effect on the system’s exfoliation. The CO_2_ generated from the carbonate stabilizes metal oxides, which increases the surface area (Equation (11)), but also fragments the particle and contributes to the stability of metal oxides. The surface manganese content is displayed, highlighting its effect on increased nucleation in the carbon matrix. The manganese is homogeneously distributed in both functionalized materials for BPP-Mn and BPS-Mn. Figure 3a–d, Table 4 [32].

MCO_3(sol)_ --~ CO_2(gas)_ + MO_(gas)_ (11)

In Figure 4, two amorphous halo patterns of the cellulose are observed with a wide band around 2θ = 25°, characteristic of activated carbon. For BPS, a peak at 29.5° is observed, which can be related to the existence of inorganic components including SiO_2_. The presence of manganese carbonate and surface Mn oxide is evidenced in the diffraction patterns, confirming the thermal effect on the formation of oxides and crystallization of the excess metal salt on the surface for BPS-Mn. Alternatively, in the presence of adsorbed surface water, these systems can promote hydroxide formation on the material’s surface (Equation (12)) due to the influence of carbonate in the structure. This leads to the negative surface charge residual, as indicated in Table 4.

MCO_3(sol)_ + H_2_O_(gas)_ --~ CO_2(gas)_ + M(OH)_2_ (12)

Figure 5a,b shows the adsorption and degradation processes of all materials concerning ACE. The pyrolyzed BPS material exhibited a synergistic effect, functioning as a carbocatalyst. However, the incorporation of metal ions into the system enhanced the adsorption of ACE. The electrochemical behavior, represented by the oxidation peak associated with manganese, was inhibited by ACE, suggesting the formation of a stabilized coordination compound, which in turn reduced the carbocatalytic activity. Lignin is the strongest component due to its highly aromatic structure of phenylpropane units in BPS concerning BPP as reported in the literature and observed in electrochemical analyses, evidencing vanillin. When lignin is pyrolyzed, the β-O-4 bonds break, forming a series of free radicals, which repolymerize to produce bio-oil, syngas, and biochar. To 500 °C phenolic compounds such as vanillin favor the coordination bonds between carbonaceous material-Mn:PMS: ACE, in high grade concerning materials with low lignin content as potato peel [33] Figure 6a–c,e–g. On the other hand, the carbonate/bicarbonate ions are strong scavengers of both hydroxyl and sulfate radicals, with high reaction rate constants, which may explain this effect, low carbocatalysis in the presence of metal carbonate (Equations (13) and (14)) [34] as depicted in Figure 6d,h.

•OH + CO_3_^2−^ → CO_3_^•−^ + OH^−^ (13)

SO_4_^•−^ + CO_3_^2−^ → CO_3_^•−^ + SO_4_^2−^ (14)

Table 5 shows that only the treatment carried out with the most active material (BPS) for degradation exhibits an increase in pH after treatment. This behavior suggests heterolytic cleavage of peroxide bond in PMS, activated by transfer electronic since carbonaceous material, Equation (15) [25].

HSO_5_^−^ + 2e^−^ → OH^−^ + SO_4_^•−^ (15)

On the other hand, Figure 8a confirms the formation of sulfate or hydroxyl radicals during the carbocatalytic process for BPS when methanol is employed as a scavenger. However, $O_{2}^{•-}$ radicals also contribute to in breaking down ACE. It is suggested that BPS is reduced through interaction with PMS, as shown in Equation (16). This happens because PMS is activated by the carbon-based material in BPS, due to its ability to transfer energy. The impedance study, shown in Figure 8b, supports this by highlighting the role of oxidation products of lignin [35]. The RAMAN spectra (Figure 6c) show the use of sp2 hybridized active sites, which aid the breakdown process after treatment.

BPS + 2HSO_5_^−^ + 1e^−^ → BPS (reduced) + O_2_^•−^ + 2HSO_4_^−^ (16)

**Scheme 1:**
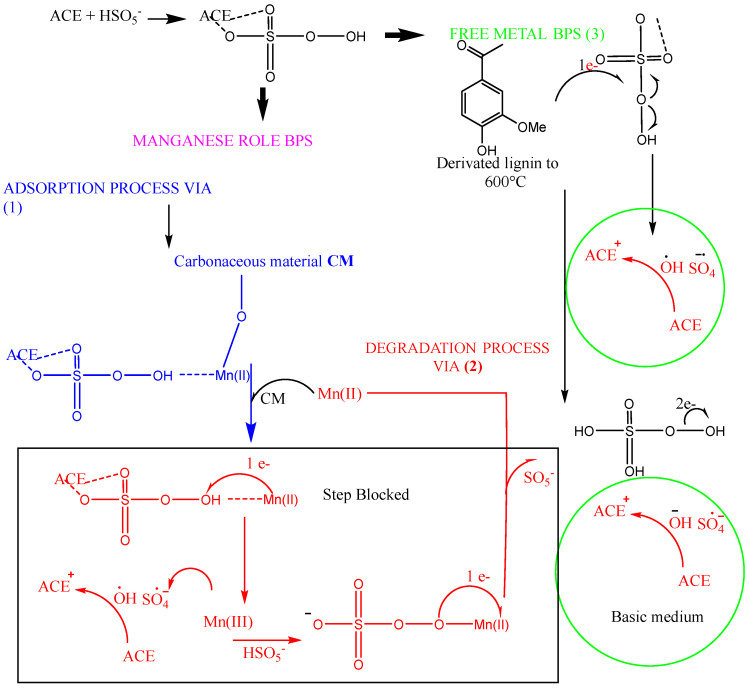
Mechanism proposed BPS and its modification with manganese.

As illustrated in Scheme 1, the proposed mechanism for ACE removal involves three key contributions: (1) the role of manganese in BPS, which aids in removal through adsorption, and (2) removal via degradation. The manganese, in forms like MnO or MnCO_3_, is supported by the carbon matrix, facilitating the adsorption of acetaminophen by forming a coordination complex between the carbon matrix, Mn, PMS, and ACE. This reduces the carbocatalytic process in BPS and minimizes the contribution to peroxo bond cleavage, while carbonate ions act as radical scavengers. In contrast, pyrolyzed BPS materials without manganese (3) show that the carbon matrix itself significantly affects drug removal, possibly due to the presence of phenolic groups (lignin derivates) that persist at 500 °C, enabling the electron transfer process to PMS. It also shows a blockage in the carbocatalysis process due to the cleavage of the peroxo bond in PMS assisted by the transition metal. This is mainly because the electrochemical profile lacks oxidation peaks of new manganese species that would confirm this degradation pathway. However, it is highlighted that BPS with manganese reduces its carbocatalytic capacity, possibly due to the presence of carbonate ions acting as scavengers of hydroxyl and sulfate radicals [36,37,38].

On other hand, previous studies have reported that the incorporation of transition metals, such as manganese and iron, onto carbonaceous materials enables the development of adsorbent and catalysts enhanced activity [39,40]. The metal oxide has also been employed to improve the adsorption characteristics of biochar. The biochar enriched with MnO_2_ displayed significantly greater adsorption capacity for methylene blue dye compared to the unmodified biochar. The maximum reported adsorption capacity was 248.96 mg/g, with a removal rate of 99.73%, in contrast to 234.65 mg/g and 94.0% for the unaltered biochar [41]. For catalytic systems, the metal enables additional active sites for redox reactions and the promotion of electron transfer between the catalyst and reactants, which facilitates the efficient activation of PMS and the generation of ROS for oxidation reactions [42]. Researches evaluated reduced graphene with Mn and Fe_2_O_4_ in increased degradation of pollutants and PMS activation (process-denominated carbocatalysis). Furthermore, the adsorption process with MnFe_2_O_4_ and MnFe_2_O_4_-graphene reached ~10% of orange II removal. The transition metal oxides as the manganese (Mn) can activate oxidants [43,44]. However, Mn is not found as a metal but as oxides, silicates, and carbonates [45]. Among them, the manganese oxides (MnO_X_) have attracted great interest because low toxicity, various forms with various oxidation states (II, III, IV, and VII), and their capacity for PMS or PDS activation in a heterogeneous system [46,47]. The literature has reported that MnO_2_ [48], Mn_2_O_3_ [49,50], and Mn_3_O_4_ can activate PMS and PDS due to redox properties between different valence states of Mn. Among the mentioned oxides MnO_2_ is the manganese oxide most studied including those amorphous and crystalline structures (α-, γ-, β-, and δ-MnO_2_). Mn-containing catalysts include direct oxidation of compounds, electron transfer, pollutants oxidation by free radicals (SO_4_^•−^, HO^•^, O_2_^•−^) [46], and non-radical by singlet oxygen (^1^O_2_) oxidation. As reported, there are few cases where manganese is incorporated into the structure as carbonate. Likewise, the implications of using the carbonaceous matrix as a support or as a source of oxygenated groups available for coordination and activation of the peroxide bond are not widely explored.

## 5. Conclusions

Manganese metal salts interact differently with carbonaceous matrices. The role of manganese in carbocatalytic processes was evaluated using two raw materials with differing lignin content. It was determined that the transition metal can facilitate the adsorption of the drug targeted for degradation. Also combined with its counterion, acts as a radical scavenger, thereby hindering the degradation process. In materials with higher lignin content exposed to 500 °C, phenolic groups are more likely to coordinate with the metal center, enabling interactions with PMS and ACE that enhance adsorption. In such cases, a balance exists between adsorption and the metal center’s ability to transfer electrons to PMS for activation, as seen with the banana pseudostem. Conversely, in materials with lower lignin content, manganese shows limited adsorption capacity and no carbocatalytic activity. While electron-donating oxygenated groups formed from the oxidation of cellulose and hemicellulose can stabilize coordination compounds, their impact is peripheral and significantly less effective compared to the phenolic groups derived from lignin. In consequence, pyrolyzed material derived from banana pseudostem demonstrated superior carbocatalytic performance. This compared to its counterpart from potato peel, a difference attributed to their distinct structural properties. This study demonstrates how lignocellulosic materials can be repurposed for the degradation of emerging contaminants and provides insight into the carbocatalysis process in the presence of peroxymonosulfate.

## Figures and Tables

**Table 1:** Compositional differences in the cell wall of the potato peel and the banana pseudostem.

Component	Species	
	Potato Peel (%)	Banana Pseudostem (%)
Lignin content	1–5	10–15
Cellulose Content	40–60	30–50
Hemicellulose content	30–20	20–25

**Table 2:** Carbocatalyst used to remove Acetaminophen.

Oxidant Agent	Material	Degradation (%) ACE	ROS Generate	Dosage on ACE Removal	Time (min)
IO_3_^−^	Carbon-doped boron nitride [23].	90.8%	^1^O_2_ O_2_^•−^	30–60 μM	8
PMS PDS (peroxidisulphate)	SFBC_800_ possesses intrinsic N and P heteroatoms, SFBC (*Sargassum fusiforme* biochar carbon) [24].	98.70%	O_2_^•−^ •OH SO_4_^•−^	[SFBC_800_] = 100 mg/L	45
PMS	Fe/HTCN-3/Vis [25].	51.9% 99.9%	^•^OH SO_4_^•−^	0.2 g/L 0.8 g/L	60
PMS	Mn/N co-doped biochar (Mn@NBC-X) [26].	79.6%	CO_3_^•−^	20 mg/L	10

**Table 3:** Textural properties of materials.

Material	Pore Volume (cm^3^)		Pore Quantity (%)		Pore Dimension (Å)		Yield (%*w*/*w*)	Especific Surface Area (S_BET,_ m^2^/g)
	Micro-Sized Pore	Meso-Sized Pore	Micro-Sized Pore	Meso-Sized Pore	Micro-Sized Pore	Meso-Sized Pore		
PP	0.002	0.006	77	23	9.5	25.8	1.95	8.9
BPP	0.003	0.014	14.6	84.4	18.3	22.3	6.19	17.3
BPP-Mn	0.115	0.029	87.0	13.0	8.3	23.3	3.53	264.9
PS	0.002	0.002	89.0	11.0	9.2	20.3	3.24	23.3
BPS	0.005	0.008	38.5	61.5	17.3	22.3	3.87	17.0
BPS-Mn	0.111	0.028	79.8	20.2	9.3	23.3	3.64	252.1

**Table 4:** Surface properties and and % Mn (EDS detection).

Material	Mn (%)	Charge Capability (mV)
BPS	5.55	−2.9
BPS-Mn	95.79	−18.3
BPP	0.17	21.4
BPP-Mn	92.62	−11.0

**Table 5:** Monitoring pH before and after treatment for BPS, BPS-Mn, BPP, and BPP-Mn against ACE degradation.

Material	pH Initial	pH Final 50 min
BPS	6.1	10.8
BPS-Mn	6.1	7.9
BPP	6.1	5.5
BPP-Mn	6.1	5.2

## Data Availability

There is no additional data beyond what is reported in this article.

## Supplementary Materials

The accompanying information is available for download at: https://www.mdpi.com/article/10.3390/polym16233316/s1. Figure S1 displays the chromatographic profile of BPS-Mn during the treatment against ACE.

## References

[1] Torres L.A.Z., Woiciechowski A.L., Tanobe V.O.d.A., Karp S.G., Lorenci L.C.G., Faulds C., Soccol C.R. (2020). Lignin as a potential source of high-added value compounds: A review. J. Clean. Prod..

[2] Bajwa D.S., Pourhashem G., Ullah A.H., Bajwa S.G. (2019). A concise review of current lignin production, applications, products, and their environment impact. Ind. Crops Prod..

[3] Machida M., Mochimaru T., Tatsumoto H. (2006). Lead(II) adsorption onto the graphene layer of carbonaceous materials in aqueous solution. Carbon.

[4] Patiño Y., Díaz E., Ordóñez S. (2014). Performance of different carbonaceous materials for emerging pollutants adsorption. Chemosphere.

[5] Álvarez-Torrellas S., Rodríguez A., Ovejero G., García J. (2016). Comparative adsorption performance of ibuprofen and tetracycline from aqueous solution by carbonaceous materials. Chem. Eng. J..

[6] Chen W.H., Naveen C., Ghodke P.K., Sharma A.K., Bobde P. (2023). Co-pyrolysis of lignocellulosic biomass with other carbonaceous materials: A review on advance technologies, synergistic effect, and future prospectus. Fuel.

[7] Romero-Hernandez J.J., Paredes-Laverde M., Silva-Agredo J., Mercado D.F., Ávila-Torres Y., Torres-Palma R.A. (2024). Pharmaceutical adsorption on NaOH-treated rice husk-based activated carbons: Kinetics, thermodynamics, and mechanisms. J. Clean. Prod..

[8] Sepelev I., Galoburda R. (2015). Industrial potato peel waste application in food production: A Review. Res. Rural Dev..

[9] Chauhan A., Islam F., Imran A., Ikram A., Zahoor T., Khurshid S., Shah M.A. (2023). A review on waste valorization, biotechnological utilization, and management of potato. Food Sci. Nutr..

[10] Daimary N., Eldiehy K.S.H., Boruah P., Deka D., Bora U., Kakati B.K. (2022). Potato peels as a sustainable source for biochar, bio-oil and a green heterogeneous catalyst for biodiesel production. J. Environ. Chem. Eng..

[11] Ahmad T., Danish M. (2018). Prospects of banana waste utilization in wastewater treatment: A review. J. Environ. Manag..

[12] Mohiuddin A., Saha M.K., Hossian M.S., Ferdoushi A. (2014). Usefulness of Banana (*Musa paradisiaca*) Wastes in Manufacturing of Bio-products: A Review. Agriculturists.

[13] Zhang Y., Chen P., Liu S., Fan L., Zhou N., Min M., Cheng Y., Peng P., Anderson E., Wang Y. (2017). Microwave-assisted pyrolysis of biomass for bio-oil production. Pyrolysis.

[14] Şerbǎnescu C. (2014). Kinetic analysis of cellulose pyrolysis: A short review. Chem. Pap..

[15] Meyer S., Glaser B., Quicker P. (2011). Technical, economical, and climate-related aspects of biochar production technologies: A literature review. Environ. Sci. Technol..

[16] Quimbaya-Ñañez C., Serna-Galvis E.A., Silva-Agredo J., García-Rubio I., Torres-Palma R.A., Ávila-Torres Y.P. (2023). Improvement of the Carbocatalytic Degradation of Pharmaceuticals in Water by the Use of Ultrasound Waves. Water.

[17] Quimbaya-Ñañez C., Serna-Galvis E.A., Silva-Agredo J., Huerta L., Torres-Palma R.A., Ávila-Torres Y. (2024). Mn-based material derived from industrial sawdust for the elimination of ciprofloxacin: Loss of antibiotic activity and toxicity via carbocatalysis assisted by ultrasound. J. Environ. Chem. Eng..

[18] Hayat W., Zhang Y., Huang S., Hussain I., Huang R. (2021). Insight into the degradation of methomyl in water by peroxymonosulfate. J. Environ. Chem. Eng..

[19] Vo H.N.P., Le G., Nguyen T.M.H., Bui X.-T., Nguyen K.H., Rene E.R., Vo T.D.H., Cao N.-D.T., Mohan R. (2019). Acetaminophen micropollutant: Historical and current occurrences, toxicity, removal strategies and transformation pathways in different environments. Chemosphere.

[20] Manasik N., Tony M.A. (2024). The Environmental Oxidation of Acetaminophen in Aqueous Media as an emerging Pharmaceutical Pollutant Using a Chitosan Waste-Based Magnetite Nanocomposite. Resources.

[21] Lin Y., Wu S., Yang C., Chen M., Li X. (2019). Preparation of size-controlled silver phosphate catalysts and their enhanced photocatalysis performance via synergetic effect with MWCNTs and PANI. Appl. Catal. B Environ..

[22] Lin Y. (2021). Interfacial Charge Transfer between Silver Phosphate and W_2_N_3_ Induced by Nitrogen Vacancies Enhances Removal of *β*-Lactam Antibiotics. Adv. Funct. Mater..

[23] Jiang L., Li W., Wang H., Yang J., Chen H., Wang X., Yuan X., Wang H. (2024). Non-radical activation of low additive periodate by carbon-doped boron nitride for acetaminophen degradation: Significance of high-potential metastable intermediates. J. Hazard. Mater..

[24] Bae S., Masud M.A., Annamalai S., Shin W.S. (2024). The inherent nature of N/P heteroatoms in Sargassum fusiforme seaweed biochar enhanced the nonradical activation of peroxymonosulfate for acetaminophen degradation in aquatic environments. Chemosphere.

[25] He Y., Xie F., Lan J., Xu Z., Liu Q., Hou H. (2024). Activation of peroxymonosulfate under visible-light illumination for enhanced degradation of acetaminophen by self-assembly iron phosphorus/hexagonal tubular graphite carbon nitride. Colloids Surf. A Physicochem. Eng. Asp..

[26] Sun M., Fang W., Liang Q., Xing Y., Lin L., Luo H. (2024). Efficient acetaminophen degradation via peroxymonosulfate activation by Mn/N co-doped biochar: Performance, mechanism and approach. J. Environ. Chem. Eng..

[27] Li J., Li B., Zhang X. (2002). Comparative studies of thermal degradation between larch lignin and manchurian ash lignin. Polym. Degrad. Stab..

[28] Mo Z., Li M., Sun S., Zhu R., Zhan D., Li A., Li Y., Zhang Y., Yu Q. (2024). Modeling of activated carbon and multi-scale molecular simulation of its water vapor adsorption: A review. J. Environ. Chem. Eng..

[29] Liang W., Li L., Li R., Yin Y., Li Z., Liu X., Shan S., He Y., Meng Y., Li Z. (2020). Crystal structure of impurity-free rhodochrosite (MnCO_3_) and thermal expansion properties. Phys. Chem. Miner..

[30] Nam K., Kim Y., Jo Y., Lee S.M., Kim B.G., Choi R., Choi S., Song H., Park J.T. (2012). New Crystal Structure: Synthesis and Characterization of Hexagonal Wurtzite MnO. J. Am. Chem. Soc..

[31] Lee J.H.Q., Lauw S.J.L., Webster R.D. (2016). The electrochemical study of Vanillin in acetonitrile. Electrochim. Acta.

[32] Galwey A.K., Brown M.E. (1999). Chapter 12 Decomposition of carbonates. Stud. Phys. Theor. Chem..

[33] Ovalle-Serrano S.A., Blanco-Tirado C., Combariza M.Y. (2018). Exploring the composition of raw and delignified Colombian fique fibers, tow and pulp. Cellulose.

[34] Balachandran R., Zhao M., Dong B., Brown I., Raghavan S., Keswani M. (2014). Role of ammonia and carbonates in scavenging hydroxyl radicals generated during megasonic irradiation of wafer cleaning solutions. Microelectron. Eng..

[35] Li P.H., Wei Y.M., Wu C.W., Yang C., Jiang B., Wu W.J. (2022). Lignin-based composites for high-performance supercapacitor electrode materials. RSC Adv..

[36] Wang J., Wang S. (2020). Reactive species in advanced oxidation processes: Formation, identification and reaction mechanism. Chem. Eng. J..

[37] Zhou Y., Jiang J., Gao Y., Pang S.-Y., Yang Y., Ma J., Gu J., Li J., Wang Z., Wang L.-H. (2017). Activation of peroxymonosulfate by phenols: Important role of quinone intermediates and involvement of singlet oxygen. Water Res..

[38] Kumar K., Kumar R., Kaushal S., Thakur N., Umar A., Akbar S., Ibrahim A.A., Baskoutas S. (2023). Biomass waste-derived carbon materials for sustainable remediation of polluted environment: A comprehensive review. Chemosphere.

[39] Liu X.J., Li M.F., Singh S.K. (2021). Manganese-Modified Lignin Biochar as Adsorbent for Removal of Methylene Blue. J. Mater. Res. Technol..

[40] Dong C.D., Chen C.W., Tsai M.L., Chang J.H., Lyu S.Y., Hung C.M. (2019). Degradation of 4-Nonylphenol in Marine Sediments by Persulfate over Magnetically Modified Biochars. Bioresour. Technol..

[41] Yao Y., Cai Y., Lu F., Wei F., Wang X., Wang S. (2014). Magnetic Recoverable MnFe_2_O_4_ and MnFe_2_O_4_-Graphene Hybrid as Heterogeneous Catalysts of Peroxymonosulfate Activation for Efficient Degradation of Aqueous Organic Pollutants. J. Hazard. Mater..

[42] Fu W.-X., Qin M.-Z., Niu H.-Y., Hu Q.-Y., Guo D., Chen M.-M., Niu C.-G., Huang D.-W. (2023). Insights into the High Performance of Co–Mn Oxide Supported by Carbon Fiber Frameworks for Ciprofloxacin Oxidation Process. J. Clean. Prod..

[43] Nriagu J. (2011). Environmental Pollution and Health Effects. Encyclopedia of Environmental Health.

[44] Zhou Z.G., Du H.M., Dai Z., Mu Y., Tong L.L., Xing Q.J., Liu S.S., Ao Z., Zou J.P. (2019). Degradation of Organic Pollutants by Peroxymonosulfate Activated by MnO2 with Different Crystalline Structures: Catalytic Performances and Mechanisms. Chem. Eng. J..

[45] Song Y., Jiang J., Ma J., Zhou Y., Von Gunten U. (2019). Enhanced Transformation of Sulfonamide Antibiotics by Manganese(IV) Oxide in the Presence of Model Humic Constituents. Water Res..

[46] Saputra E., Muhammad S., Sun H., Ang H.M., Tadé M.O., Wang S. (2013). Different Crystallographic One-Dimensional MnO_2_ Nanomaterials and Their Superior Performance in Catalytic Phenol Degradation. Environ. Sci. Technol..

[47] Chen L., Fu W., Hou C., Yang Y., Zhang X. (2021). A Comprehensive Assessment of Catalytic Performances of Mn_2_O_3_ Nanoparticles for Peroxymonosulfate Activation during Bisphenol a Degradation. Catalysts.

[48] Khan A., Zhang K., Sun P., Pan H., Cheng Y., Zhang Y. (2021). High Performance of the A-Mn_2_O_3_ Nanocatalyst for Persulfate Activation: Degradation Process of Organic Contaminants via Singlet Oxygen. J. Colloid Interface Sci..

[49] Wang F., Xiao M., Ma X., Wu S., Ge M., Yu X. (2021). Insights into the Transformations of Mn Species for Peroxymonosulfate Activation by Tuning the Mn3O4 Shapes. Chem. Eng. J..

[50] Saputra E., Muhammad S., Sun H., Ang H.M., Tadé M.O., Wang S. (2013). Manganese Oxides at Different Oxidation States for Heterogeneous Activation of Peroxymonosulfate for Phenol Degradation in Aqueous Solutions. Appl. Catal. B Environ..

