# Supplementary material for: Effects of Manganese Carbonate Addition on the Carbocatalytic Properties of Lignocellulosic Waste for Use in the Degradation of Acetaminophen
Source: Polymers (Basel). 2024 Nov 27;16(23):3316. doi: 10.3390/polym16233316 (PMC11644440; doi:10.3390/polym16233316)
Supplement: Supplementary file 1 [file polymers-16-03316-s001.zip › polymers-3288088-supplementary.pdf]

Supplementary information

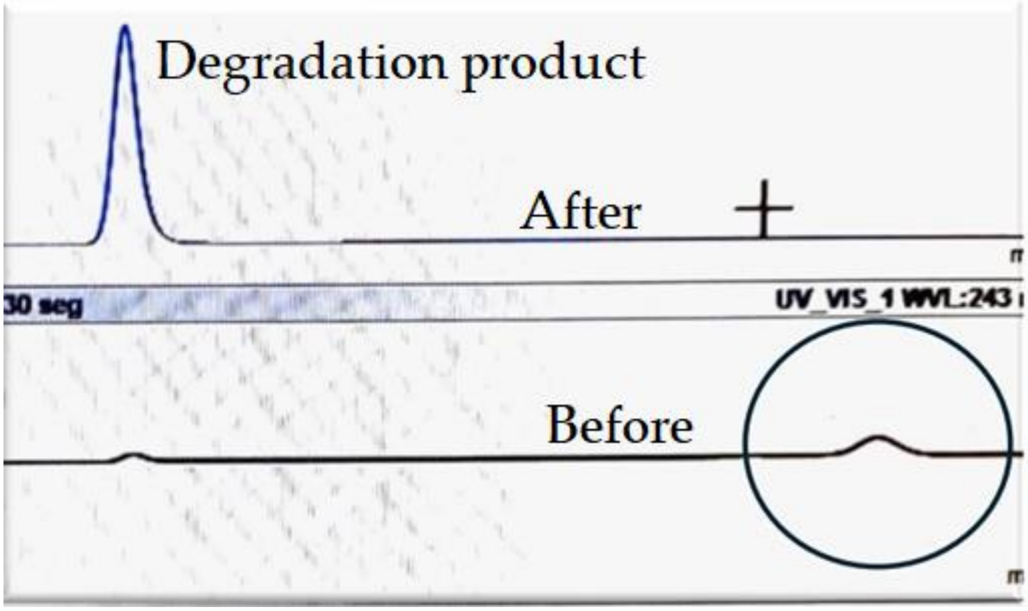

Figure S1. Performance of ACE (after and before treatment) using BPS-Mn
